# Surface profile analysis of laminated transfemoral prosthetic socket fabricated with different ratios of epoxy resin and acrylic resin

**DOI:** 10.1038/s41598-022-21990-y

**Published:** 2023-02-15

**Authors:** Nik Abdul Muiz Nik Zainuddin, Nasrul Anuar Abd Razak, Mohd Sayuti Ab Karim, Noor Azuan Abu Osman

**Affiliations:** 1 Department of Biomedical Engineering, Faculty of Engineering, Universiti Malaya, Kuala Lumpur, Malaysia; 2 Department of Mechanical Engineering, Faculty of Engineering, Universiti Malaya, Kuala Lumpur, Malaysia

**Keywords:** Biomedical engineering, Mechanical engineering

## Abstract

Acrylic and epoxy are common types of resin used in fabricating sockets. Different types of resin will affect the internal surface of a laminated socket. This paper is to determine the best combination of ratio for epoxy and acrylic resin for a laminated prosthesis socket and to evaluate the surface profile analysis of different combinations of laminated prosthetic sockets for surface roughness. Transfemoral sockets were created using various resin-to-hardener ratios of 2:1, 3:1, 3:2, 2:3, and 1:3 for epoxy resin and 100:1, 100:2, 100:3, 100:4, and 100:5 for acrylic resin. Eight layers of stockinette consisting of four elastic stockinette and four Perlon stockinette were used. A sample with a size of 4 cm × 6 cm was cut out from the socket on the lateral side below the Greater Trochanter area. The Mitutoyo Sj-210 Surface Tester stylus was run through the sample and gave the Average Surface Roughness value (Ra), Root Mean Square Roughness value (Rq), and Ten-Point Mean Roughness value (Rz). Epoxy resin shows a smoother surface compared to acrylic resin with Ra values of is 0.766 µm, 0.9716 µm, 0.9847 µm and 1.5461 µm with 3:2, 3:1, 2:1 and 2:3 ratio respectively. However, for epoxy resin with ratio 1:3, the resin does not cure with the hardener. As for acrylic resin the Ra values are 1.0086 µm, 2.362 µm, 3.372 µm, 4.762 µm and 6.074 µm with 100: 1, 100:2, 100:5, 100:4 and 100:3 ratios, respectively. Epoxy resin is a better choice in fabricating a laminated socket considering the surface produced is smoother.

## Introduction

Prosthetics devices are artificial limbs fabricated as a replacement of missing body limb^1,2^. Goals for prosthetic is to restore normal daily life activities to the user^3,4^. Different fabrication techniques available in fabricating these devices such as thermoforming and lamination^5^. Where thermoforming softened a plastic sheet and place it onto a positive cast where lamination use resin and hardener to coat the positive cast^5–7^. These process and materials induced different mechanical properties of a prosthetic socket^8^. The recommended ratio of resin to hardener for epoxy is 2:1 while for acrylic, the supplier catalogue mentioned is 100:1–3.

In terms of mechanical qualities such as ultimate tensile strength, flexural strength, and stiffness, prosthetic sockets manufactured from laminated composites have been found to be stronger than copolymers thermoplastic sockets^9–11^. The amount of vacuum pulled during construction, the degree of wet-out (saturation of resin into the reinforcement material), the type of resin, the amount of resin, and the type of fibre reinforcement can all create variations in laminated prosthetic sockets^5,12^.

The epidermis, subcutaneous tissue, blood vessels, and blood flow of the residual limb are all affected by the pressure and friction created by the movement. Reciprocal sliding friction on the skin surface would tend to break down the efficiency of the stratum corneum barrier function and induce the skin trauma^13,14^. The coefficient of friction and energy dissipation between the prosthetic socket and liner materials are both affected by surface roughness^15,16^. Most of the transfemoral participants had used either strap or suction suspension (CSS)^17^. For a transfemoral patient with suction suspension, the socket interfaces directly to the patient’s skin thus giving the impact on the skin condition.

When it comes to thermal stability, the created composites outperformed pure epoxy resin in terms of reduced degradation rate at the same temperature and higher enthalpy, proving that natural fibres reinforced epoxy composites are far superior to pure epoxy resin^18^. Acrylic had 33% higher transverse tensile strength and equivalent modulus. It had comparable longitudinal flexural strength and modulus. It had slightly lower transverse flexural strength and modulus. It exhibited superior fracture toughness and delamination resistance. Micrographs revealed microstructural ductility in acrylic and brittle fracture mechanisms in epoxy. Acrylic had a higher tan delta peak than epoxy^19,20^.

However, the difference of surface roughness of epoxy and acrylic resin are not mention in any study. Thus, the study is keen in investigating the surface roughness of both type of resin to determine the better fabrication for a prosthetic socket in term of surface roughness for better comfort.

## Method

### Materials

Materials used in this study were acrylic resin; Orthocryl Laminierharz 80:20 (617H19) (Ottobock, Inc., Duderstadt, Germany) with Ottobock hardening powder (617P37) (Ottobock, Inc., Duderstadt, Germany) as hardener, epoxy resin; Epoxen CP362 part A with hardener CP362 part B (Oriental Option Sdn Bhd, Penang, Malaysia). The polyvinylalcohol (PVA) bag was made using Ottobock PVA sheeting (616F4). Stockinette used also obtained from Ottobock which is Perlon Elastic stokinette, white (623T5 = 15) (Ottobock, Inc., Duderstadt, Germany) with width of 15 cm. Elastic stockinette was provided by Centre for Prosthetic and Orthotic Engineering (CPOE) with width also 15 cm.

### Socket fabrication

The positive cast were obtained by copying a polypropylene transfemoral socket provided from Centre for Prosthetic and Orthotic Engineering (CPOE) into a negative cast. The negative cast was then filled with Plaster of Paris (POP) slurry made by mixing POP powder and water. As the POP slurry hardened the negative cast was removed and the positive cast was modified and smoothed.

Lamination technique begin by preparing 2 polyvinylalcohol (PVA) bag according to the size of the positive cast. Lay up of 8 layers of reinforcement materials was put in between the PVA bag consisting of 4 perlon stokinette and 4 elastic stokinette. A mixture of resin and hardener ranged 600-610 g was made in a cup with different combination ratio as shown in the Table 1.

**Table 1:** Resin to Hardener ratio.

Transfemoral socket	1	2	3	4	5
Epoxy resin (resin: hardener)	2:1	3:2	2:3	3:1	1:3
Acrylic resin (resin: hardener)	100:1	100:2	100:3	100:4	100:5

The solution was then poured into the PVA bag-Reinforcement materials sandwich. Each socket was made using acrylic resin and epoxy resin under vacuum suction of less than 20% non-inductive until it is hot indicating it has cured. The laminated composite was then left overnight before finishing the socket by smoothen the edge of the socket trimline. The laminated socket was then cut according the trimline to be pull out from the positive cast. A sample cut out was taken from the lateral part of the socket 21 cm from the distal end and 3 cm from medial wall sized of 4 cm x 6 cm.

### Surface testing for laminated socket

**Figure 1:**
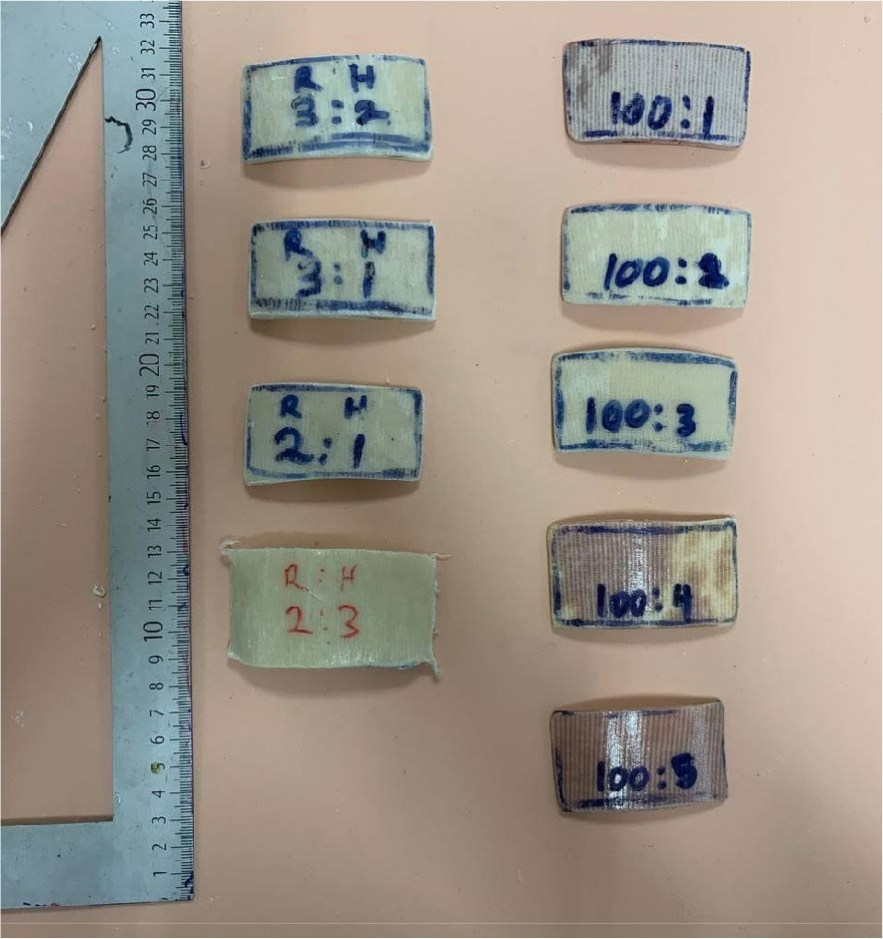
Samples cutouts.

Laminated transfemoral sockets surfaces sample cut outs of around 4 cm × 6 cm (benchmark samples) as shown in Fig. 1. A profilometer is a typical tool for determining surface roughness. A table-top contact profilometer was used to assess the surface roughness of the Pe-Lite samples (Mitutoyo SurfTest SJ-210 series)^21,22^. A retractable probe with a diamond tip stylus was included with the profilometer. The stylus had a 2.5 μm radius and was fitted with a 0.75 mN measuring force. For each surface twenty trials were conducted.

**Figure 2:**
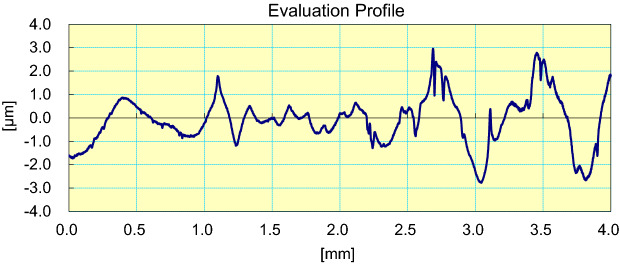
2D graph generated from the Mitutoyo Surftest SJ-210.

**Figure 3:**
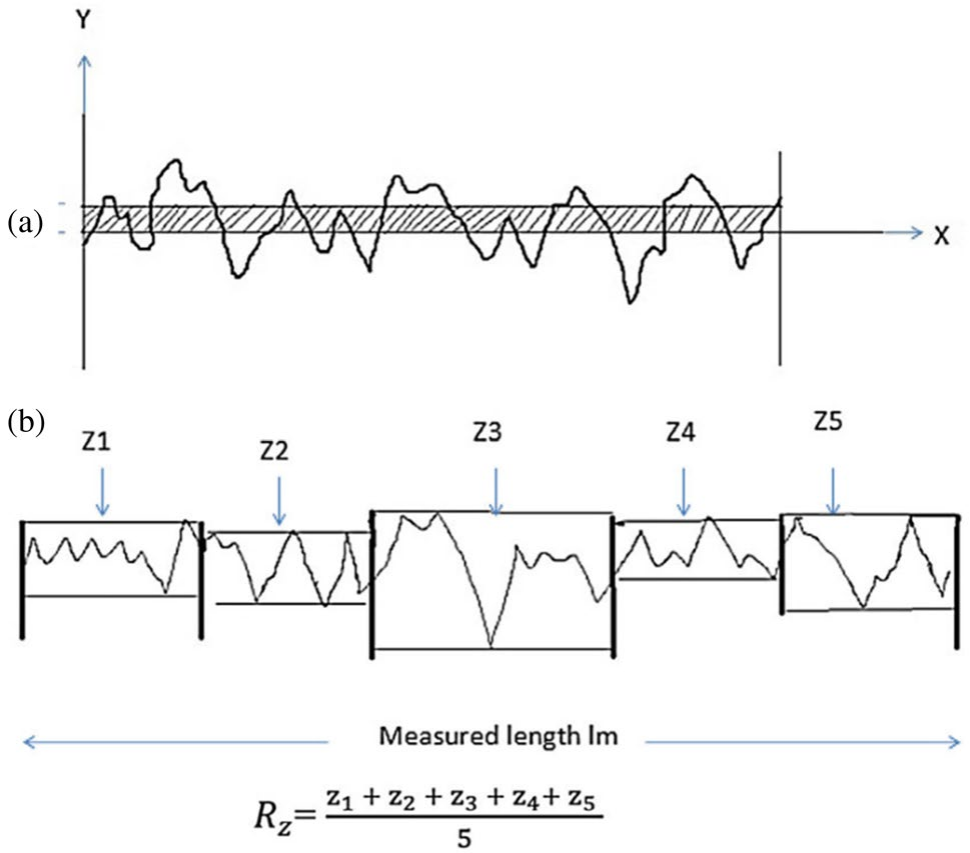
Illustration of Ra and Rz Values.

The topographical analysis was carried out with the use of a portable gadget connected to a communication software program that enabled for real-time inspection to be recorded and shown the 2D analysis graphs automatically as shown in Fig. 2. Average surface roughness (Ra), root mean square roughness (Rq), and ten-point mean roughness (Rz) were chosen as roughness parameters. Ra is obtained by measuring the mean deviation of the peaks from the centre line of trace, the centre line being established as the line above & below which there is an equal area between the Centre line and the surface trace. There is little difference between the Centre Line Average (CLA) & Root mean square (RMS) values for a given surface. It is the average of the single peak to valley heights of n number of adjoining sampling Lengths. The illustration of the this principle is shown in Fig. 3.

**Figure 4:**
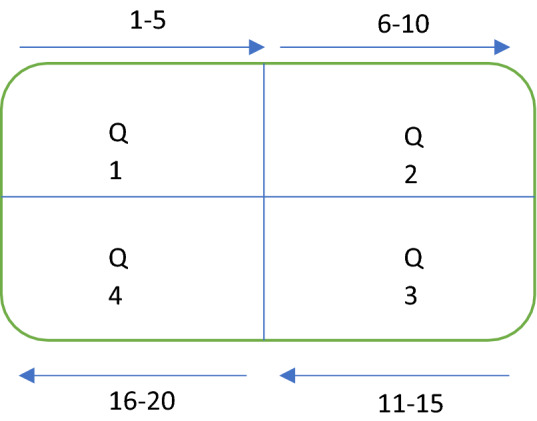
Sequences of surface testing.

Twenty trials were conducted by running the stylus onto the samples. The samples are divided into four equal segments five trial are conducted on each segment. The sequences of the trials are shown in Fig. 4. The trials started from the anterior-distal segment and ends on the anterior-proximal segment.

Lastly, to obtain the comparison data, means of all twenty trials were conducted for all three surface roughness parameters of the nine different ratios of resin to hardener.

## Result

Table 2 shows the time taken for different ratios of resin and hardener for two types of resin. The time taken for one part of epoxy resin and three part of hardener is not recorded as the mixture does not cure. Time taken for acrylic resin to cure is shorter compared to epoxy resin with maximum time taken is only 167 min while for the epoxy resin, the minimum time taken is 480 min.

**Table 2:** Cure time at different ratios of Resin.

Epoxy resin		Acrylic resin	
Ratio (resin:hardener)	Time taken (minutes)	Ratio (resin:hardener)	Time taken (minutes)
2:1	570	100:1	167
3:1	660	100:2	43
3:2	480	100:3	40
2:3	1440	100:4	30
1:3	–	100:5	34

**Figure 5:**
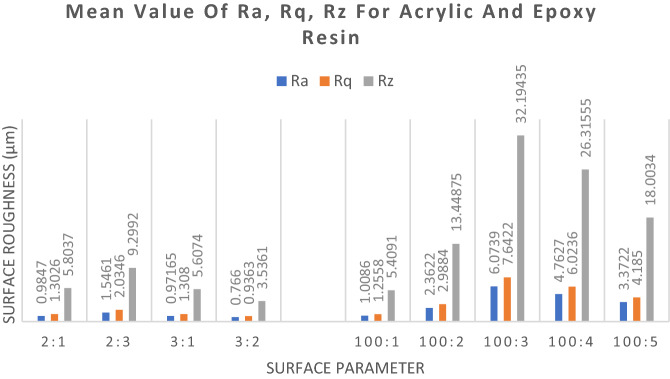
Mean Ra, Rq, Rz value of different resin and ratios.

Figure 5 shows the values for Ra, Rq and Rz values for different ratios of resin and hardener for epoxy resin and acrylic resin. Epoxy resin with ratio of 3:2, resin to hardener shows the lowest Values for all parameters. While acrylic resin with ratio of 100:3 resin to hardener shows the highest values for all parameters.

## Discussion

### Ratio versus time

Result shows that cure time depends on the amount of hardener used. More hardener will cure the composite faster^23,24^. As we can see that the cure time of for the composite increase as the amount of hardener decrease as shown in Table 2. However, the amount of hardener must not exceed the resin, this produced an uncured composite as we can see with epoxy resin to hardener of 1:3 and the same case also happened with the ratio of 2:3 where the socket takes a day to cure and the composite is soft. Thus, these ratios need to be avoided. The cure time for acrylic resin shows clearer pattern as the time taken for the composite to cure decrease as the hardener increased. The last ratio combination however exceeds the previous one by 4 min. This pattern shows the behaviour of acrylic resin where intermediate amount of hardener has different onset temperature compare to low amount and high amount of hardener^25^. Acrylic shows faster curing time as it is a thermoplastic materials whereas epoxy resin is a thermoset materials^23,26^.

### Internal surface analysis

**Figure 6:**
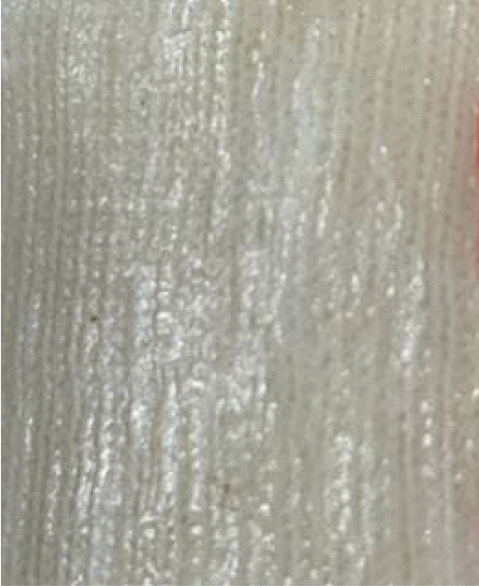
Internal surface of TF socket with 2:3 epoxy resin to hardener ratio.

For epoxy resin, the smoothest internal surface was produced by the 3:2 resin to hardener ratio followed by 3:1, 2:1 and lastly with value higher than 1, 2:3. The socket made with 1:3 resin to hardener ratio was excluded in surface testing as the composite did not cure end up in liquid state. This is due to the amount of epoxide molecules are fully reacted with hardener molecules leaving extra hardener molecule in free^23,27^. Socket made with 2:3 resin to hardener ratio has the highest Ra, Rq and Rz values compared to other epoxy socket because the socket is soft and produce visible wrinkles as in Fig. 6 which are invisible in other sockets. The epoxy groups are prone to reaction with primary amines with an increase of hardener amount. This increase the amount of time taken for the mixture to cure and result in optimum time taken for the resin to slip in the reinforcement materials, fill in the gap, minimum air bubbles formation and void and allows air bubbles to be suction out. The epoxy with a large excess of hardener has a looser epoxy network^23,24^.

As for acrylic resin, the smoothness of the internal surfaces is related to the amount of hardener used. This may be affected by the cure time of the composite. As the composite slowly curing, the resin-hardener mixture was allowed to flow more freely creating less void as compare to other ratios that have faster cure time. The smoothest surface was given by the ratio of 100:1, resin to hardener, with Ra value of 1.0086 µm as referred to Fig. 5. As discussed, the 100:1 ratio take the longest time to cure,167 min, thus it is expected to produce the smoothest surface. 100:2 resin to hardener ratio reduced the cure time significantly to just 43 min, this also doubled the Ra value to 2.3622 µm as compare to 100:1 ratio. Ratio of 100:3 shows the highest value of all three parameters Ra, Rq and Rz, this is a bit peculiar as the next ratio which is 100:4 and 100:5 have lower parameters values. This is due to the initiator contents are associated with a small number of free radicals disturbing monomers and, therefore, also responsible for a propagation based on a lower number of growing chains with greater length^25,26^. High initiator content sample, on the other hand, the propagation is based on the competitive growth of many short forming chains because of the greater number of free radicals avail-able. Consequently, the polymer content is increased and can promote the so called gel effect^25,28^ This suggesting the instability in with the 100:3 ratio but the mixture become more stable with low and high amount of hardener.

The way the related surface contacts behave regarding friction is essential. Surface roughness plays a significant impact in determining friction since these variables are related to one another^29^. Important information about how the skin interacts with different surfaces is revealed by friction studies. The reduction areas of a positive cast will exert the maximum pressure in the case of a prosthetic socket^30^ since these regions are in charge of regulating residual limb movement in the socket. Additionally, the patients' walking speed will have a varied impact on the friction^31^.

ANOVA one-way test in Table 3 revealed that between epoxy resin data do not reject the null hypothesis of equality of means for all ratios with p-value = 1.00 but there is rejection of the null hypothesis of equality in means between epoxy and acrylic resin. The socket made from epoxy resin indicates the rejection of the null hypothesis of equality in means to the socket made from acrylic resin with ratio 100:1 as the *p*-value = 1 this also applies to acrylic resin with 100:2 ratio especially with epoxy Resin with ratio 2:3. Meanwhile, 100:3 to 100:5 acrylic resin ratios show a very strong significance as *p*-value is calculated to be less than 0.001. In between the acrylic resin socket, a few ratios are statistically similar to the other such as 100:1 with 100:2, 100:2 with 100:5, 100:3 with 100:4, and lastly 100:4 with 100:5.

**Table 3:** *p*-value between parameters of different ratios of resin and hardener.

(I) Samples_ratio	(J) Samples_ratio	Sig	(I) Samples_ratio	(J) Samples_ratio	Sig
			100:1	2:1	1.000
2:1	2:3	1.000		2:3	1.000
	3:1	1.000		3:1	1.000
	3:2	1.000		3:2	1.000
	100:1	1.000		100:2	0.231
	100:2	0.312		100:3	< 0.001
	100:3	< 0.001		100:4	< 0.001
	100:4	< 0.001		100:5	< 0.001
	100:5	< 0.001	100:2	2:1	0.312
2:3	2:1	1.000		2:3	1.000
	3:1	1.000		3:1	0.270
	3:2	1.000		3:2	0.033
	100:1	1.000		100:1	0.231
	100:2	1.000		100:3	< 0.001
	100:3	< 0.001		100:4	< 0.001
	100:4	< 0.001		100:5	1.000
	100:5	0.069	100:3	2:1	< 0.001
3:1	2:1	1.000		2:3	< 0.001
	2:3	1.000		3:1	< 0.001
	3:2	1.000		3:2	< 0.001
	100:1	1.000		100:1	< 0.001
	100:2	0.270		100:2	< 0.001
	100:3	< 0.001		100:4	1.000
	100:4	< 0.001		100:5	< 0.001
	100:5	< 0.001	100:4	2:1	< 0.001
3:2	2:1	1.000		2:3	< 0.001
	2:3	1.000		3:1	< 0.001
	3:1	1.000		3:2	< 0.001
	100:1	1.000		100:1	< 0.001
	100:2	0.033		100:2	< 0.001
	100:3	< 0.001		100:3	1.000
	100:4	< 0.001		100:5	0.169
	100:5	< 0.001	100:5	2:1	< 0.001
				2:3	0.069
				3:1	< 0.001
				3:2	< 0.001
				100:1	< 0.001
				100:2	1.000
				100:3	< 0.001
				100:4	0.169

## Conclusion

As the result displayed, Epoxy resin comes superior in term of smoothness of the internal surface but it takes longer to fabricate. Epoxy resin of 3:2 resin to hardener ratio gives the smoothest surface, however the instructed epoxy resin ratio is 2:1 but it does not give the smoothest surface. The difference between 2:1 and 3:2 is not that significant with only 0.218 µm thus further on the difference of other mechanical properties will determine the best ratio to use in fabricating a laminated socket. Smoother surface of transfemoral socket will give patients more comforts and can promote rehabilitation process with longer use of prosthesis (Supplement Information).

## Supplementary Information

Supplementary Information.

## Data Availability

The datasets used and/or analysed during the current study available from the corresponding author on reasonable request.
